# Gene expression analyses in breast cancer epidemiology: the Norwegian Women and Cancer postgenome cohort study

**DOI:** 10.1186/bcr1859

**Published:** 2008-02-13

**Authors:** Vanessa Dumeaux, Anne-Lise Børresen-Dale, Jan-Ole Frantzen, Merethe Kumle, Vessela N Kristensen, Eiliv Lund

**Affiliations:** 1 Institute of Community Medicine, University of Tromsø, 9037 Tromsø, Norway; 2 Department of Genetics, Institute for Cancer Research, Norwegian Radium Hospital, Rikshospitalet University Hospital, Oslo, Norway; 3 Faculty Division The Norwegian Radium Hospital, University of Oslo, Norway; 4 Institute for Clinical Medicine, University of Tromsø, 9037 Tromsø, Norway

## Abstract

**Introduction:**

The introduction of high-throughput technologies, also called -omics technologies, into epidemiology has raised the need for high-quality observational studies to reduce several sources of error and bias.

**Methods:**

The Norwegian Women and Cancer (NOWAC) postgenome cohort study consists of approximately 50,000 women born between 1943 and 1957 who gave blood samples between 2003 and 2006 and filled out a two-page questionnaire. Blood was collected in such a way that RNA is preserved and can be used for gene expression analyses. The women are part of the NOWAC study consisting of 172,471 women 30 to 70 years of age at recruitment from 1991 to 2006 who answered one to three questionnaires on diet, medication use, and lifestyle. In collaboration with the Norwegian Breast Cancer Group, every NOWAC participant born between 1943 and 1957 who is admitted to a collaborating hospital for a diagnostic biopsy or for surgery of breast cancer will be asked to donate a tumor biopsy and two blood samples. In parallel, at least three controls are approached for each breast cancer case in order to obtain blood samples from at least two controls per case. The controls are drawn at random from NOWAC matched by time of follow-up and age. In addition, 400 normal breast tissues as well as blood samples will be collected among healthy women participating at the Norwegian Mammography Screening program at the Breast Imaging Center at the University Hospital of North-Norway, Tromsø.

**Results:**

The NOWAC postgenome cohort offers a unique opportunity (a) to study blood-derived gene expression profiles as a diagnostic test for breast cancer in a nested case-control design with adjustment for confounding factors related to different exposures, (b) to improve the reliability and accuracy of this approach by adjusting for an individual's genotype (for example, variants in genes coding for hormone and drug-metabolizing and detoxifying enzymes), (c) to study gene expression profiles from peripheral blood as surrogate tissue to biomonitor defined exposure (for example, hormone) and its association with disease risk (that is, breast cancer), and (d) to study gene variants (single nucleotide polymorphisms and copy number variations) and environmental exposure (endogenous and exogenous hormones) and their influence on the incidence of different molecular subtypes of breast cancer.

**Conclusion:**

The NOWAC postgenome cohort combining a valid epidemiological approach with richness of biological samples should make an important contribution to the study of the etiology and system biology of breast cancer.

## Introduction

Observational epidemiologic data are used as major criteria to categorize any chemicals or lifestyle factors as carcinogenic. This is an inherent part of the carcinogen classification as carried out by the World Health Organization through its cancer research center at the International Agency for Research on Cancer in Lyon, France [1]. The burden of cancer still increases and the term 'human carcinogen' is important for cancer prevention since it forms much of the basis for public health initiatives like programs for stopping the smoking epidemic, advice about medication use, and workplace safety.

At first, a prospective cohort study consisted of exposure information given by questionnaires and participant follow-up, either passive through different registers or active by repeated interviews. Collection of biological samples, mainly from peripheral blood, for exposure or biomarker measurements became part of many epidemiological research designs in the 1970s. Plasma and serum can be used to study proteins and metabolites, and with recent large-scale approaches, the total proteome and metabolome can be analyzed. In addition, the availability of germline DNA gives the opportunity for genotyping, followed by gene-environment and gene-gene interaction studies. Today, hundreds of thousands of probes spotted either on a glass slide or on beads allow whole-genome scans of single nucleotide polymorphisms (SNPs) and copy number variations (CNVs) for each individual. About a decade ago, new inventions opened up a third level of studies investigating gene expression at the RNA level from peripheral blood and tumor tissue by microarray technology. It should be noted that, due to the effect of RNases, which degrade RNA unless stabilized into a specific buffer, standard biobanks do not provide high-quality mRNA.

The introduction of high-throughput technologies, also called -omics technologies, into epidemiology has raised the need for high-quality observational studies to reduce several sources of error and bias [2,3]. This is related to the selection of study populations, technical challenges, and the necessary size of the studies. To overcome some of these challenges and to be able to take full advantage of the new technologies, the Norwegian Women and Cancer (NOWAC) postgenome cohort study was created with a biobank that gives the possibilities for RNA and DNA whole-genome scans of both peripheral blood and breast tissue. Here, we present the unique design of the NOWAC postgenome cohort which will enable us to fully investigate all aspects of the relationship between exposures and breast cancer by giving us the opportunity to look at genetic predisposition (CNVs or SNPs), gene expression (RNA and microRNA), gene products (proteome) and their metabolites (metabolome), and information given by questionnaire in relation to breast cancer risk, stratified by molecular markers of tumor subtypes.

## Materials and methods

**Figure 1:**
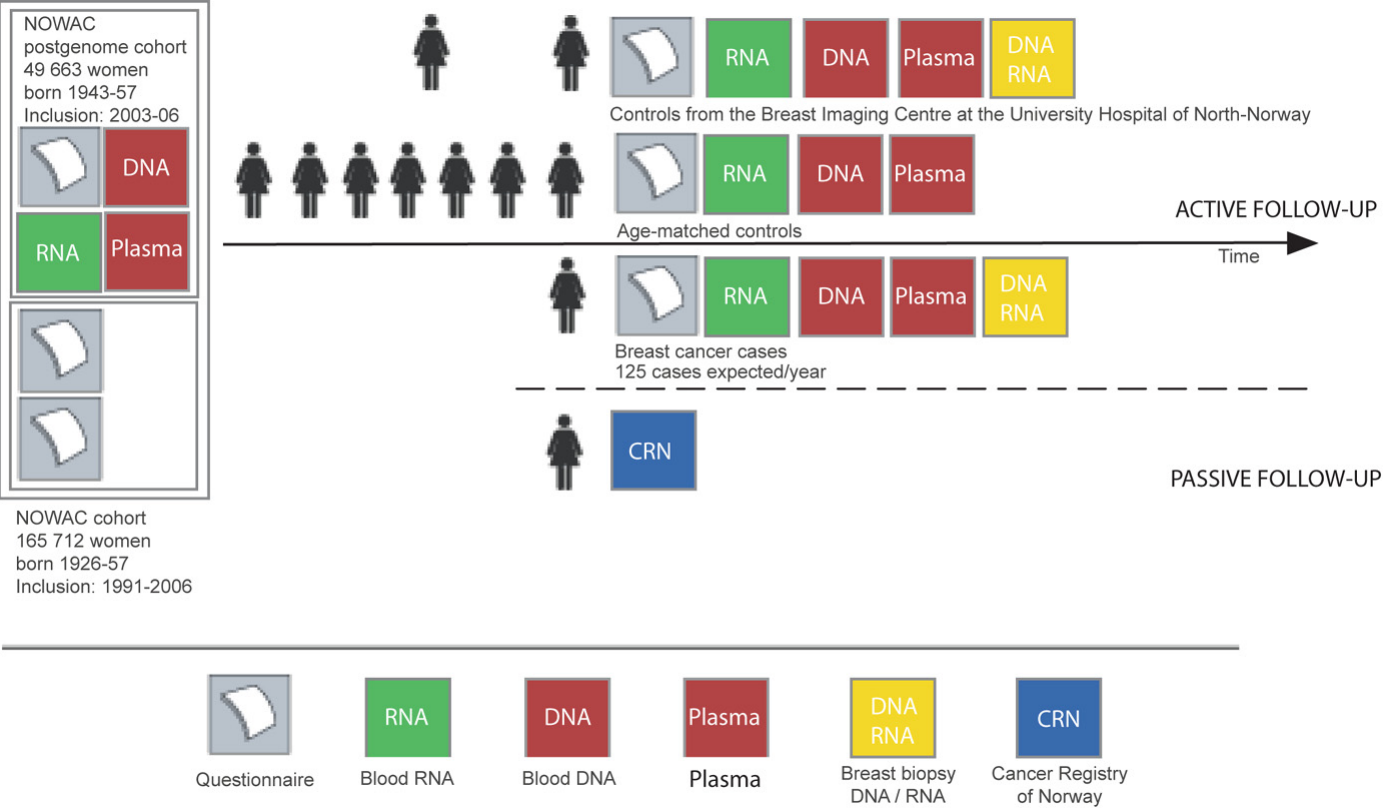
Study design of the Norwegian Women and Cancer (NOWAC) postgenome cohort study.

The NOWAC postgenome cohort consists of approximately 50,000 women born between 1943 and 1957 who gave blood samples between 2003 and 2006 (Figure 1). The women are part of the NOWAC study consisting of 172,471 women 30 to 70 years of age at recruitment from 1991 to 2006 ([4] and Figure 1 of [5]). Of the 172,471 women participating today, 148,536 were born between 1943 and 1957. The whole cohort consists of random samples from all counties in Norway, but in Northern Norway (three counties), Rogaland, and Oslo, all women in this age range were invited to participate. The overall response rate is close to 72.3%. It decreases slightly with age and varies by region with a higher response rate in North-Norway [6]. The participants have answered one to three four-page questionnaires of core variables: use of hormonal treatments, reproductive history, ages at menarche and menopause, smoking, physical activity, alcohol consumption, anthropometry, socioeconomic status, screening for breast cancer, breast cancer in the family, sunbathing habits and pigmentation, and self-reported diseases. A major part of the questionnaires has, in addition, four pages asking for detailed information on dietary habits. In the introduction letter, they were informed that they could be asked for a blood donation. Notably, BRCA (Breast Cancer gene) status was not specifically asked for but family history for breast cancer was provided by questionnaire as listed above. In the clinic, women with strong family history are referred for counselling to cancer genetic specialists by their local physician.

Women included in the postgenome NOWAC study were sampled at random from the whole cohort in series of 500 women each. Each woman received a package with an information folder explaining the purpose of the study with the specific items related to the informed consent. The women were asked to visit their family doctor or any other authorized place for blood drawing and to take with them the necessary equipment that they had received: a special glass tube for conservation of mRNA in a buffer (PAXgene™ Blood RNA System; PreAnalytiX GmbH, Hombrechtikon, Switzerland), a glass tube with citrate, needles, and a prepaid envelope for high-priority post for shipping the biological samples. At the time of blood sampling, they had to fill out a two-page questionnaire with information on current use of hormonal treatments and other pharmaceutical therapeutics (over-the-counter and prescription), dietary supplements, smoking, and height and weight. The blood samples then were sent by overnight mail to the Institute of Community Medicine at the University of Tromsø, Norway. On arrival, the citrate glass tube was centrifuged and 'buffy-coated' and two tubes of plasma were frozen at -80°C. The PAXgene™ tubes were frozen directly at -80°C as recommended by the manufacturer.

### Passive follow-up

Information on cancer cases among NOWAC postgenome cohort members is sought by yearly linkage to the Norwegian Cancer Register based on the unique person number [7] (so far, until the end of 2005). As of 31 December 2005, there were 2,237 incident breast cancer cases among the first 102,410 women included in NOWAC. The Norwegian Cancer Register is building a nationwide database reporting treatment and follow-up information for breast cancer. Possible linkage to the cancer register database is indicated in the informed consent form, but approval from the ethical committee to do this linkage is a prerequisite.

### Active follow-up: blood and breast tissue collection from cases and controls

Norway has around 436,338 women born between 1943 and 1957 [8], of whom 148,536 (34.4%) participate in NOWAC. At present, 11 of the largest Norwegian hospitals participate in collecting blood and tumor tissue from incident breast cancer cases. In collaboration with the Norwegian Breast Cancer Group, every woman born between 1943 and 1957 who is admitted to a collaborating hospital for a diagnostic biopsy or for surgery of breast cancer will be asked whether she participates in the NOWAC study. If she answers yes, she will be asked to donate a tumor biopsy collected in RNALater and two blood samples, one collected into PAXgene™ and another in a citrate tube for RNA, DNA, protein, and metabolite analyses (Figure 1). RNALater provides a simple, safe, and effective method for prospectively acquiring and processing breast core needle biopsies for gene expression studies [9]. If she does not remember her earlier participation in NOWAC, information is sought in the NOWAC files based on name and birth year. NOWAC participants then are asked to sign a new informed consent form and to answer a short questionnaire eliciting information mainly on use of medications. In the same manner as in the postgenome cohort, biological samples are mailed overnight to Tromsø.

In parallel, at least three controls are approached for each breast cancer case in order to obtain blood samples from at least two controls per case. The controls are drawn at random from the same original series but matched by time of follow-up and age. During the first 8 months of active follow-up, we received 73 biological samples from incident breast cancer cases or an estimated number of 110 per year.

The Norwegian Breast Cancer Screening program invites all women 50 to 69 years of age to mammography every second year. The period of the next 6 years of the NOWAC cohort fairly corresponds to the screening cohort and this gives an opportunity to collect breast tissue from controls among the participants in NOWAC. The aim is to collect 400 samples of normal breast tissue as well as blood samples among healthy women participating in the Norwegian Mammography Screening program at the Breast Imaging Center at the University Hospital of North-Norway, Tromsø (Figure 1). The women are asked to sign an informed consent form and to answer the same questionnaires as the cases. The tissue and blood samples are managed in the same manner as the cases. So far, about 60 tissue samples have been collected. It is planned to collect 10 samples a week.

### Validity

The external validity of NOWAC as a representative sample of the Norwegian female population has been verified in several methodological analyses and found to be acceptable [6]. Studies of the internal validity, including reliability, have been undertaken for the dietary questions [10-12], menopausal status, and use of hormonal replacement therapy [13], whereas validation of physical activity is ongoing.

### Biobank and database

The Medical Faculty at the University of Tromsø is responsible for the maintenance of the biobank with quality and security control. The data are held by the NOWAC research team at the Institute of Community Medicine, Medical Faculty, University of Tromsø, Norway. The University of Tromsø has the legal rights to this database.

### Ethical issues

We have received approval from the Regional Committee for Medical Research Ethics for the collection and storing of questionnaire information, blood samples, and tumor tissue. All women have filled out an informed consent form for later linkages to national registers. The informed consent formula explicitly mentions that the blood samples can be used for gene expression analyses as well as large-scale genotyping. All data are stored and handled according to the permission given by the Norwegian Data Inspectorate. The Directorate of Health and Social affairs (SHD) has given us an exemption from the confidentiality of information in national registers. Before every use of the biological material, a request will be sent to the regional ethical committee for Northern-Norway. Use of biological material requires permission according to laws pertaining to biotechnology and gene technology, both of which are administered by the SHD.

### Improvement in technology and analysis

#### Globin RNA processing methods for genome-wide transcriptome analysis from whole blood

The use of the PAXgene™ Blood RNA System offers advantages for the shipping of blood samples by direct preservation of RNA. However, excess globin mRNA originating mainly from lysed immature reticulocytes in the stabilized samples has been shown to adversely impact gene expression profiling using microarrays [14]. In a pilot study, we assessed whether RNA from whole blood with or without globin reduction gives the most robust and sensitive results to detect small gene expression changes in response to hormone therapy (HT) exposure [15]. Overall, no sign of higher sensitivity in the detection of gene expression changes using the Applied Biosystems Microarray System (Applied Biosystems, Foster City, CA, USA) was observed after globin reduction, although in previous studies using Affymetrix GeneChip arrays (Affymetrix, Santa Clara, CA, USA), it appeared to be beneficial [16-20]. We concluded from this that the appropriate RNA processing method should be chosen according to the microarray platform to be used. However, this will pose a problem regarding standardization of the methods since globin-reduced and non-globin-reduced approaches produce distinct gene expression profiles which are not directly comparable [15,19].

White blood cells have been defined as the most transcriptionally active cells in blood and may give the most sensitive gene expression profiles in response to defined factors [21]. However, a PAXgene™ tube with relative compromised sensitivity for global gene expression profiling, but with the advantage of stabilizing RNA in such a large-scale study, may be suitable to study some exposure or health status. For others, it may be beneficial to perform additional fractionation in a nested case-control design to isolate only the cells belonging to a subtype.

#### Choice of microarray platform

In our exploratory research investigating blood gene expression profiles according to hormone exposure in women [22], we initially used the Human 1A oligo arrays (22K) produced by Agilent Technologies, Inc. (Santa Clara, CA, USA). This two-color array platform required cohybridization of a sample and a standard reference, so the relative expression level for each individual gene can be readily normalized and related across multiple experiments. The commercially available Universal Human Reference RNA from Stratagene (La Jolla, CA, USA) [23] is designed to provide a baseline measurement of every gene, although this common reference does not have any biological meaning. We also encountered hybridization problems with Cy5 dye degradation due to the presence of ozone [24]. After a special washing step with a stabilization and drying solution recommended by the producer, red and green average signals were approximately at the same level, but with lower mean intensity (V. Dumeaux, A-L. Børresen-Dale, E. Lund, unpublished data). To be noted, Agilent Technologies, Inc., has developed a new dual-channel workflow as well as a one-channel protocol option that are supposed to overcome some of these issues.

In our following study, we used the Applied Biosystems Human Genome Survey oligo-microarray platform, a one-color system based on chemiluminescence [15]. The results were satisfying and indicated that smaller variations were reliably detected. However, the throughput of the Applied Biosystems platform is rather low and therefore not ideal for large-scale epidemiological studies. The bead array system produced by Illumina, Inc. (San Diego, CA, USA) seems to be a cost-effective alternative, hopefully as sensitive and reliable without the need for globin reduction, but this should be evaluated before conducting blood gene expression analyses in future studies of the NOWAC cohort.

### Research designs

#### 1. Blood-derived gene expression profiles as diagnostic test for breast cancer adjusted for exposure confounder effect

Mammography is by far the most commonly used method for early detection of breast cancer in women 50 to 69 years of age. However, the sensitivity, specificity, and cost of mammography screening have been debated for many years. Tumors less than 5 mm in size are, in general, difficult to detect by mammography, and some types of breast cancer like lobular cancer are often missed. In addition, it is difficult to interpret mammograms from women with dense breast tissue, a common feature of younger women [25,26]. Also, some tumors may develop too rapidly to be identified at the most treatable stage by mammography screening every second year [27]. Many women are also diagnosed with *in situ *cancer (ductal carcinoma *in situ*, DCIS), and it is widely discussed whether all women with DCIS will develop a malignant tumor and whether too many are unnecessarily treated. Finally, sensitivity of the mammography screening is comparatively high (>75%) while spontaneous regression of many subclinical cancerous tumors may not be uncommon [28]. This is important because the effects of overdiagnosis extend beyond living with the diagnosis of cancer and include adverse effects of treatment for breast cancer (surgery, endocrine therapy, chemotherapy, and radiotherapy).

There is a growing evidence that by analyzing the changes in gene activity in sensor cells (like blood cells), it might be possible to provide information on whether tumor cells are present elsewhere in the body (for instance, in the breast). Some studies have found that the use of peripheral blood cells for transcriptome analysis was valuable in assessing disease-associated [29-33] and drug-response-related [34] gene signatures. The search for gene expression profiles in blood-based tests for early diagnosis of breast cancer as an added test in relation to mammographic screening is ongoing based on case-control design and using women with dense mammograms as controls [33]. If a dense mammogram is part of the causal chain of breast cancer, the use of such controls may reduce the sensitivity of the test because controls could express genes partly as a result of an early carcinogenic process. On the other hand, the signatures developed using such controls may help identify cancer cases in the group of women with uncertain mammograms.

Adjusting the analysis of gene expression profiles for confounders may increase the sensitivity of the diagnostic profile since exposure risk factors for breast cancer could partly explain the expression of genes in the cases. In the NOWAC cohort, current users of HT had a twofold increase in breast cancer risk compared with never users [35] and small changes in blood-derived gene expression profiles for HT users were observed compared with non-HT users [22]. Thus, gene expression changes in a diagnostic test for breast cancer without adjustment for HT use could be due, at least partly, to different prevalences of HT use among cases and controls. In this way, the NOWAC postgenome cohort offers a unique opportunity to study blood-derived gene expression profiles as a diagnostic test for breast cancer in a nested case-control design with adjustment for confounding factors related to different exposures. In addition, given the prospective design of the study with blood sampling prior to diagnosis, blood gene expression profiles can also be studied as a risk predictor for breast cancer.

#### 2. Influences of an individual's genotype on breast cancer risk

Gene expression changes adjusted for environmental exposure may be insufficient to make an accurate diagnosis or prediction. Breast cancer, like all types of cancer, is considered to be a genetic disease in the sense that both germline and somatic mutations may be the cause of tumor initiation and tumor development. Genetic determinants (for example, variants in genes coding for hormone and drug-metabolizing and detoxifying enzymes) may need to be incorporated to improve the reliability and accuracy of these approaches. New technologies allow whole-genome scans in unrelated cases and controls in genome-wide association (GWA) studies in the search for common genetic variants associated with disease risk. Recently, by means of this approach, novel variants in five genes were found to slightly increase susceptibility to breast cancer [36,37]. The increased risks associated with these alleles are relatively small, but, on the other hand, these susceptibility alleles are very common (for example, approximately 14% of the UK population are homozygous for the risk allele of the SNP rs2981582 in *FGFR2*). Thus, other mutations that contribute similarly to breast cancer susceptibility may be found using GWA studies. As additional susceptibility alleles are identified, a combination of such alleles together with other breast cancer risk factors may be relevant to implement into clinical practice.

Although there is little doubt that the novel susceptibility markers produced from such highly powered studies are true, the mechanism by which they cause the susceptibility remains unravelled. SNPs in the recently discovered susceptibility genes may also exert their effect through the expression of their genes in tumors, giving rise to the various breast cancer molecular subtypes [38]. Below in section 4, we further discuss how the NOWAC postgenome cohort gives a unique opportunity to stratify patients by their tumor molecular subtypes, which may give more power to the classical case-control studies to identify genes of no or borderline significance for breast cancer as such, but which may be more highly penetrant for certain subclasses.

A strong influence of SNPs in master regulator genes on gene expression in tumors has already been detected [39], and the NOWAC study will give us the opportunity to explore the SNP effect on gene expression in blood and tissue in both healthy and disease states. In an exploratory approach, whole-genome SNPs scans that cover more than 100,000 SNPs (100 K Illumina array) have been conducted for the NOWAC women used in our pilot study for HT profiling [22] in order to explore the effect of SNP profiles on blood gene expression in both HT users and nonusers.

#### 3. Blood as surrogate tissue to conduct gene expression profiles

The ability to investigate the biological mechanisms and obtain diagnostic and prognostic information about a target tissue (for example, breast tissue) by using easily accessible surrogate tissues and fluids (for example, blood) has significant and far-reaching implications for basic and clinical research. Surrogate analysis is not a new concept, but the development of -omic technologies has broadened both the range of tissues that can be examined and the number of targets that can be analyzed in a single experiment. One of the greatest challenges is the interpretation and appropriate use of all -omic data obtained from target and surrogate tissues.

We hypothesized that gene expression changes in blood caused by different exposure and combined with an individual's genotype could be used prospectively to identify an elevated breast cancer risk. Also, gene expression profiles associated with a disease state may become apparent in such prospective studies. This can provide potential gene markers of susceptibility and response to exposure which can be used for preventive strategies or could be of potential mechanistic interest. Ideal genomic biomarkers for exposure would be RNA species with wide dynamic range, which increased or decreased in expression in surrogate tissues proportionally to the dose level.

Blood is currently the most practical choice for surrogate tissue. The relationship between gene expression in target (that is, breast) and surrogate (that is, blood) tissues, however, must be better established. Results from Rockett and colleagues [40] have demonstrated that in rats many genes are coexpressed in peripheral blood cells and the uterus. Although alterations in transcriptional profiles of peripheral blood of patients with cancer may not share the same identity with those observed in the primary tumor, such patterns nonetheless would be of tremendous physiological relevance and bear obvious diagnostic value in the assessment of this disease. In the NOWAC study, questionnaires about exposure (in particular, hormone exposure), blood, and breast tissue biopsy from both healthy women and breast cancer cases offer a unique opportunity to investigate these critical questions.

#### 4. Genetic and environmental influences on the incidence of different molecular subtypes of breast cancer

Breast cancer is a heterogeneous disease, and it is likely that different risk factors and susceptibility factors give rise to different types of breast cancer. Gene expression profiles have the power of capturing the complexities of tumors and can be used to portray a tumor's detailed phenotype in its unique context as a basis for an improved diagnostic description or a new molecular taxonomy of breast cancer [41]. Tumors now can be grouped according to their expression profiles, and the influence of gene variants (SNPs and CNVs) and environmental exposure (endogenous and exogenous hormones) on the development of different expression 'motifs' of breast cancer can be studied [42]. Thus, low-penetrant breast cancer gene variants and gene-environment interactions that are otherwise difficult to identify in classical case-control studies may appear to be more highly penetrant for certain subclasses and therefore identifiable. Finding susceptibility loci may assist us also in establishing the causes of different molecular subtypes.

It is critical to use common criteria for molecular profiling of tumors to avoid the daunting problem of differences caused by artifacts. Five subtypes of breast tumors characterized by specific expression patterns using hierarchical clustering [41,43,44] were associated with significant differences in overall and relapse-free survival and have been found repeatedly in other expression data sets showing the robustness of this classification [43,45].

Similarly, to elucidate gene-environment interactions, the availability of accurate data pertaining to environmental influences is critical. This permits the investigation of the underlying mechanisms of cancer as the basis for developing new strategies for the intervention or prevention of cancer. Similarly, gene-gene interactions may play an important role in cancer susceptibility. Recent work suggests that, when gene-environment or gene-gene interactions exist, accounting for these in the whole-genome analysis will improve the probability of retaining causal loci in the second and subsequent stages of multistage designs [46].

## Discussion

The perspective of the postgenome NOWAC cohort is to merge a new research field to epidemiology by adding gene expression profiles both from peripheral blood and tumor tissue to the prospective design, in our case restricted to breast cancer and healthy controls. Uncertainty remains about the use of gene expression profiles from peripheral blood as surrogate tissue to biomonitor defined exposure (that is, hormone) and its association with disease risk (that is, breast cancer). However, we believe that gene expression profiling in human populations could assist us in (a) measuring exposition and defining outcome, (b) understanding mode of action, (c) understanding the etiology of environmentally induced disease, and (d) improving risk assessment methods and models.

Misclassification of exposure and outcome is an important source of bias in epidemiologic studies, and most study designs provide little opportunity to focus on biological mechanisms underlying the exposure-disease relationship. Gene expression profiling provides an opportunity to move beyond traditional approaches to exposure assessment (for example, environmental studies based on one chemical agent at a time) and to outcome assessment (for example, histological type in cancer). This comprehensive view of exposure and outcome is needed to define complex exposure-disease relationships and the interactions between genetic and environmental factors in human disease.

Genetic markers of disease susceptibility can influence exposure effects and therefore are important to consider in risk assessments. However, these markers may offer both promise and peril for individual and population risk assessments. The promise is for a more refined assessment of risk through the identification of gene-gene and gene-environment interactions and also for focusing on prevention and control programs for high-risk individuals. The perils include ethical and social issues, including stigmatization and discrimination. One misconception is to believe that removing a susceptible individual from the exposure without reducing exposure opportunities reduces risk, whereas this may not be so since polymorphisms in low-penetrant genes often require exposure to environmental factors to be effective, meaning that the effect is attributable to the gene-environment interaction, not to the genetic trait itself [47].

Due to the huge number of genotyped SNPs, genome-wide scans are faced with a major statistical problem even when a single underlying biological hypothesis is being tested. Thus, sample availability and current genotyping costs almost certainly will limit the first genome-wide scans to detect main effects under the hypothesis that a single etiologic entity is involved [48]. However, each cancer may represent multiple tumor types within a single histopathologic entity and it will be critical to use common criteria for molecular profiling of tumors to avoid the problem of differences caused by artifacts in expression profiling [48]. Thus, although there is still work to be done to identify robust molecularly defined subtypes for tumors at most sites, the availability of tissues in which to stratify subtypes by molecular markers is a valuable adjunct to initial scans. One purpose of NOWAC was to create a large prospective cohort in order to collect the necessary number of cases required for subtype-specific hypotheses. The implications of these findings for cancer prevention, detection, and treatment, and whether all this information will lead to actionable strategies, are unclear. However, we can hope that identifying genes in biological pathways will give us important information in cancer causation.

The use of a matched nested case-control design in a large prospective representative cohort will reduce the possible deleterious effect of differential recall bias for the exposure information and possible systematic errors of selection bias. Most other studies have not collected cases and controls from the same study base, which is very important for the validity of the comparison between cases and controls. The controls should reflect the exposure levels in the population in which the cases appear.

## Conclusion

The NOWAC postgenome cohort was designed for research in functional genomics. The combination of a valid epidemiological approach together with richness of biological samples should make an important contribution to the study of the system biology of breast cancer.

## Abbreviations

CNV = copy number variation; DCIS = ductal carcinoma *in situ*; GWA = genome-wide association; HT = hormone therapy; NOWAC = Norwegian Women and Cancer (study); SHD = the Directorate of Health and Social affairs; SNP = single nucleotide polymorphism.

## Competing interests

The authors declare that they have no competing interests.

## Authors' contributions

EL is the principal investigator of the NOWAC study. VD, ALBD, and EL participated in the design and elaboration of the postgenome NOWAC study, conducted the pilot studies, and drafted the manuscript. JOF and MK organized the collection of breast tissue throughout the Norwegian Breast Cancer Group. VNK participated in the design and elaboration of the genotyping studies. All authors read and approved the final manuscript.

## References

[B1] Cogliano V, Grosse Y, Baan R, Straif K, Secretan B, El Ghissassi F, WHO International Agency for Research on Cancer (2005). Carcinogenicity of combined oestrogen-progestagen contraceptives and menopausal treatment. Lancet Oncol.

[B2] Ransohoff DF (2005). Bias as a threat to the validity of cancer molecular-marker research. Nat Rev Cancer.

[B3] Wild CP (2005). Complementing the genome with an 'exposome': the outstanding challenge of environmental exposure measurement in molecular epidemiology. Cancer Epidemiol Biomarkers Prev.

[B4] Lund E, Dumeaux V, Braaten T, Hjartåker A, Engeset D, Skeie G, Kumle M (2008). Cohort profile: The Norwegian Women and Cancer Study – NOWAC – Kvinner og kreft. Int J Epidemiol.

[B5] The Norwegian Women and Cancer study, NOWAC. http://www.nowac.uit.no.

[B6] Lund E, Kumle M, Braaten T, Hjartaker A, Bakken K, Eggen E, Gram TI (2003). External validity in a population-based national prospective study – the Norwegian Women and Cancer Study (NOWAC). Cancer Causes Control.

[B7] Lunde AS, Lundeborg S, Lettenstrom GS, Thygesen L, Huebner J (1980). The person-number systems of Sweden, Norway, Denmark, and Israel. Vital Health Stat 2.

[B8] Cancer Registry of Norway (2006). http://www.kreftregisteret.no/frame.htm?english.htm.

[B9] Ellis M, Davis N, Coop A, Liu M, Schumaker L, Lee RY, Srikanchana R, Russell CG, Singh B, Miller WR, Stearns V, Pennanen M, Tsangaris T, Gallagher A, Liu A, Zwart A, Hayes DF, Lippman ME, Wang Y, Clarke R (2002). Development and validation of a method for using breast core needle biopsies for gene expression microarray analyses. Clin Cancer Res.

[B10] Slimani N, Kaaks R, Ferrari P, Casagrande C, Clavel-Chapelon F, Lotze G, Kroke A, Trichopoulos D, Trichopoulou A, Lauria C, Bellegotti M, Ocké MC, Peeters PH, Engeset D, Lund E, Agudo A, Larrañaga N, Mattisson I, Andren C, Johansson I, Davey G, Welch AA, Overvad K, Tjønneland A, Van Staveren WA, Saracci R, Riboli E (2002). European Prospective Investigation into Cancer and Nutrition (EPIC) calibration study: rationale, design and population characteristics. Public Health Nutr.

[B11] Parr CL, Veierod MB, Laake P, Lund E, Hjartaker A (2006). Test-retest reproducibility of a food frequency questionnaire (FFQ) and estimated effects on disease risk in the Norwegian Women and Cancer Study (NOWAC). Nutr J.

[B12] Hjartåker A, Andersen LF, Lund E (2007). Comparison of diet measures from a food-frequency questionnaire with measures from repeated 24-hour dietary recalls. The Norwegian Women and Cancer Study. Public Health Nutr.

[B13] Waaseth M, Bakken K, Dumeaux V, Olsen KS, Rylander C, Figenschau Y, Lund E (2008). Hormone replacement therapy use and plasma levels of sex hormones in the Norwegian Women and Cancer Postgenome Cohort – a cross-sectional analysis. BMC Womens Health.

[B14] Debey S, Schoenbeck U, Hellmich M, Gathof BS, Pillai R, Zander T, Schultze JL (2004). Comparison of different isolation techniques prior gene expression profiling of blood derived cells: impact on physiological responses, on overall expression and the role of different cell types. Pharmacogenomics J.

[B15] Dumeaux V, Lund E, Børresen-Dale AL (2008). Comparison of globin RNA processing methods for genome-wide transcriptome analysis from whole blood. Biomarkers in Medicine.

[B16] (2003). Affymetrix technical note: globin reduction protocol: a method for processing whole blood RNA samples for improved array results. http://www.affymetrix.com/support/technical/technotes/blood2_technote.pdf.

[B17] (2006). Ambion TechNotes 13(3): improved methods for gene expression profiling from blood samples. http://www.ambion.com/techlib/tn/133/7.html.

[B18] Debey S, Zander T, Brors B, Popov A, Eils R, Schultze JL (2006). A highly standardized, robust, and cost-effective method for genome-wide transcriptome analysis of peripheral blood applicable to large-scale clinical trials. Genomics.

[B19] Liu J, Walter E, Stenger D, Thach D (2006). Effects of globin mRNA reduction methods on gene expression profiles from whole blood. J Mol Diagn.

[B20] (2005). Expression analysis technical note: globin RNA reduction in blood samples. http://www.expressionanalysis.com/pdf/Globin_TechNote_2005.pdf.

[B21] Li L, Ying L, Naesens M, Xiao W, Sigdel T, Hsieh S, Martin J, Chen R, Liu K, Mindrinos M, Davis R, Sarwal M (2008). Interference of globin genes with biomarker discovery for allograft rejection in peripheral blood samples. Physiol Genomics.

[B22] Dumeaux V, Johansen J, Borresen-Dale AL, Lund E (2006). Gene expression profiling of whole-blood samples from women exposed to hormone replacement therapy. Mol Cancer Ther.

[B23] Novoradovskaya N, Whitfield ML, Basehore LS, Novoradovsky A, Pesich R, Usary J, Karaca M, Wong WK, Aprelikova O, Fero M, Perou CM, Botstein D, Braman J (2004). Universal Reference RNA as a standard for microarray experiments. BMC Genomics.

[B24] Fare TL, Coffey EM, Dai H, He YD, Kessler DA, Kilian KA, Koch JE, LeProust E, Marton MJ, Meyer MR, Stoughton RB, Tokiwa GY, Wang Y (2003). Effects of atmospheric ozone on microarray data quality. Anal Chem.

[B25] Hindle WH, Davis L, Wright D (1999). Clinical value of mammography for symptomatic women 35 years of age and younger. Am J Obstet Gynecol.

[B26] Lannin DR, Harris RP, Swanson FH, Edwards MS, Swanson MS, Pories WJ (1993). Difficulties in diagnosis of carcinoma of the breast in patients less than fifty years of age. Surg Gynecol Obstet.

[B27] Barnes DM, Bartkova J, Camplejohn RS, Gullick WJ, Smith PJ, Millis RR (1992). Overexpression of the c-erbB-2 oncoprotein: why does this occur more frequently in ductal carcinoma in situ than in invasive mammary carcinoma and is this of prognostic significance?. Eur J Cancer.

[B28] Zahl PH, Maehlen J (2005). Model of outcomes of screening mammography: spontaneous regression of breast cancer may not be uncommon. BMJ.

[B29] Twine NC, Stover JA, Marshall B, Dukart G, Hidalgo M, Stadler W, Logan T, Dutcher J, Hudes G, Dorner AJ, Slonim DK, Trepicchio WL, Burczynski ME (2003). Disease-associated expression profiles in peripheral blood mononuclear cells from patients with advanced renal cell carcinoma. Cancer Res.

[B30] Tang Y, Nee AC, Lu A, Ran R, Sharp FR (2003). Blood genomic expression profile for neuronal injury. J Cereb Blood Flow Metab.

[B31] Gladkevich A, Kauffman HF, Korf J (2004). Lymphocytes as a neural probe: potential for studying psychiatric disorders. Prog Neuropsychopharmacol Biol Psychiatry.

[B32] Achiron A, Gurevich M, Friedman N, Kaminski N, Mandel M (2004). Blood transcriptional signatures of multiple sclerosis: unique gene expression of disease activity. Ann Neurol.

[B33] Sharma P, Sahni NS, Tibshirani R, Skaane P, Urdal P, Berghagen H, Jensen M, Kristiansen L, Moen C, Sharma P, Zaka A, Arnes J, Sauer T, Akslen LA, Schlichting E, Børresen-Dale AL, Lönneborg A (2005). Early detection of breast cancer based on gene-expression patterns in peripheral blood cells. Breast Cancer Res.

[B34] Burczynski ME, Twine NC, Dukart G, Marshall B, Hidalgo M, Stadler WM, Logan T, Dutcher J, Hudes G, Trepicchio WL, Strahs A, Immermann F, Slonim DK, Dorner AJ (2005). Transcriptional profiles in peripheral blood mononuclear cells prognostic of clinical outcomes in patients with advanced renal cell carcinoma. Clin Cancer Res.

[B35] Bakken K, Alsaker E, Eggen AE, Lund E (2004). Hormone replacement therapy and incidence of hormone-dependent cancers in the Norwegian Women and Cancer study. Int J Cancer.

[B36] Easton DF, Pooley KA, Dunning AM, Pharoah PD, Thompson D, Ballinger DG, Struewing JP, Morrison J, Field H, Luben R, Wareham N, Ahmed S, Healey CS, Bowman R, Meyer KB, Haiman CA, Kolonel LK, Henderson BE, Le Marchand L, Brennan P, Sangrajrang S, Gaborieau V, Odefrey F, Shen CY, Wu PE, Wang HC, Eccles D, Evans DG, Peto J, SEARCH collaborators (2007). Genome-wide association study identifies novel breast cancer susceptibility loci. Nature.

[B37] Hunter DJ, Kraft P, Jacobs KB, Cox DG, Yeager M, Hankinson SE, Wacholder S, Wang Z, Welch R, Hutchinson A, Wang J, Yu K, Chatterjee N, Orr N, Willett WC, Colditz GA, Ziegler RG, Berg CD, Buys SS, McCarty CA, Feigelson HS, Calle EE, Thun MJ, Hayes RB, Tucker M, Gerhard DS, Fraumeni JF, Hoover RN, Thomas G, Chanock SJ (2007). A genome-wide association study identifies alleles in FGFR2 associated with risk of sporadic postmenopausal breast cancer. Nat Genet.

[B38] Nordgard SH, Johansen FE, Alnæs GI, Naume B, Børresen-Dale AL, Kristensen VN (2007). Genes harbouring susceptibility SNPs are differentially expressed in the breast cancer subtypes. Breast Cancer Res.

[B39] Kristensen VN, Edvardsen H, Tsalenko A, Nordgard SH, Sørlie T, Sharan R, Vailaya A, Ben-Dor A, Lønning PE, Lien S, Omholt S, Syvänen AC, Yakhini Z, Børresen-Dale AL (2006). Genetic variation in putative regulatory loci controlling gene expression in breast cancer. Proc Natl Acad Sci USA.

[B40] Rockett JC, Burczynski ME, Fornace AJ, Herrmann PC, Krawetz SA, Dix DJ (2004). Surrogate tissue analysis: monitoring toxicant exposure and health status of inaccessible tissues through the analysis of accessible tissues and cells. Toxicol Appl Pharmacol.

[B41] Sørlie T, Perou CM, Tibshirani R, Aas T, Geisler S, Johnsen H, Hastie T, Eisen MB, van de Rijn M, Jeffrey SS, Thorsen T, Quist H, Matese JC, Brown PO, Botstein D, Eystein Lønning P, Børresen-Dale AL (2001). Gene expression patterns of breast carcinomas distinguish tumor subclasses with clinical implications. Proc Natl Acad Sci USA.

[B42] Millikan RC, Newman B, Tse CK, Moorman PG, Conway K, Smith LV, Labbok MH, Geradts J, Bensen JT, Jackson S, Nyante S, Livasy C, Carey L, Earp HS, Perou CM (2007). Epidemiology of basal-like breast cancer. Breast Cancer Res Treat.

[B43] Sorlie T, Tibshirani R, Parker J, Hastie T, Marron JS, Nobel A, Deng S, Johnsen H, Pesich R, Geisler S, Demeter J, Perou CM, Lønning PE, Brown PO, Børresen-Dale AL, Botstein D (2003). Repeated observation of breast tumor subtypes in independent gene expression data sets. Proc Natl Acad Sci USA.

[B44] Perou CM, Sørlie T, Eisen MB, van de Rijn M, Jeffrey SS, Rees CA, Pollack JR, Ross DT, Johnsen H, Akslen LA, Fluge O, Pergamenschikov A, Williams C, Zhu SX, Lønning PE, Børresen-Dale AL, Brown PO, Botstein D (2000). Molecular portraits of human breast tumours. Nature.

[B45] Langerod AA, Zhao HH, Borgan OO, Nesland JJ, Bukholm II, Ikdahl TT, Kaaresen RR, Borresen-Dale AL, Jeffrey SS (2007). TP53 mutation status and gene expression profiles are powerful prognostic markers of breast cancer. Breast Cancer Res.

[B46] Evans DM, Marchini J, Morris AP, Cardon LR (2006). Two-stage two-locus models in genome-wide association. PLoS Genet.

[B47] Vineis P, Schulte P, McMichael AJ (2001). Misconceptions about the use of genetic tests in populations. Lancet.

[B48] Hunter DJ, Thomas G, Hoover RN, Chanock SJ (2007). Scanning the horizon: what is the future of genome-wide association studies in accelerating discoveries in cancer etiology and prevention?. Cancer Causes Control.

